# Do not feed the wildlife: associations between garbage use, aggression, and disease in banded mongooses (*Mungos mungo*)

**DOI:** 10.1002/ece3.2343

**Published:** 2016-07-25

**Authors:** Bonnie Fairbanks Flint, Dana M. Hawley, Kathleen A. Alexander

**Affiliations:** ^1^ Department of Biological SciencesVirginia TechBlacksburgVirginia; ^2^ Department of Fish and Wildlife ConservationVirginia TechBlacksburgVirginia; ^3^ Center for African Resource: AnimalsCommunities, and Land use (CARACAL)KasaneBotswana

**Keywords:** Human‐modified landscapes, provisioning, refuse, supplementation, urban wildlife, waste management, wildlife management

## Abstract

Urbanization and other human modifications of the landscape may indirectly affect disease dynamics by altering host behavior in ways that influence pathogen transmission. Few opportunities arise to investigate behaviorally mediated effects of human habitat modification in natural host–pathogen systems, but we provide a potential example of this phenomenon in banded mongooses (*Mungos mungo*), a social mammal. Our banded mongoose study population in Botswana is endemically infected with a novel *Mycobacterium tuberculosis* complex pathogen, *M. mungi*, that primarily invades the mongoose host through the nasal planum and breaks in the skin. In this system, several study troops have access to human garbage sites and other modified landscapes for foraging. Banded mongooses in our study site (*N* = 4 troops, ~130 individuals) had significantly higher within‐troop aggression levels when foraging in garbage compared to other foraging habitats. Second, monthly rates of aggression were a significant predictor of monthly number of injuries in troops. Finally, injured individuals had a 75% incidence of clinical tuberculosis (TB) compared to a 0% incidence in visibly uninjured mongooses during the study period. Our data suggest that mongoose troops that forage in garbage may be at greater risk of acquiring TB by incurring injuries that may allow for pathogen invasion. Our study suggests the need to consider the indirect effects of garbage on behavior and wildlife health when developing waste management approaches in human‐modified areas.

## Introduction

Urbanization and other forms of anthropogenic landscape alteration are increasing worldwide, generating a need to understand the direct and indirect influence of human‐modified environments on disease emergence and dynamics in humans and wildlife (Bradley and Altizer 2007; Gottdenker et al. 2014). Anthropogenic landscape modification can cause changes in population density, behavior, and/or physiology of hosts, pathogens, and vectors important for disease dynamics. In some cases, it is simply a “numbers game”: urbanization and other forms of human modification to the landscape can alter the number of species potentially involved in pathogen dilution or transmission (Keesing et al. 2010) or the population sizes or densities of competent hosts or vectors, which leads to a concomitant change in contact rates and pathogen transmission potential (Bradley and Altizer 2007; Acosta‐Jamett et al. 2011; Shapiro et al. 2012; Johnson et al. 2013). Alteration of wildlife host behavior is a less considered but likely widespread effect of human habitat modification on disease dynamics that is particularly interesting due to the potential for behavior to influence both host exposure to pathogens and/or susceptibility to disease (Hawley et al. 2011; Fairbanks and Hawley 2012). These behavioral changes add a layer of complexity to the effects of urbanization on disease dynamics because they involve a multitude of possible indirect effects which have not yet been well explored in free‐living host–pathogen systems.

Landscape modification can have significant effects on the spatial and temporal dynamics of resource availability, influencing the behavior of wildlife populations living in these human‐modified landscapes (McKinney 2006). Humans often discard plant material, animal carcasses, and food waste at garbage sites, which can become a predictable, nonseasonal, and highly concentrated source of food for wildlife. Thus, garbage sites are a specific component of human‐altered landscapes that might affect disease dynamics not only through garbage‐induced changes in host immunity or host demography, but also through changes in host behavior such as contact rates (Becker et al. 2015). For example, when two similarly sized populations of raccoons (*Procyon lotor*) were supplemented with equal amounts but different distributions of food, raccoons aggregated at clumped food resources but did not aggregate at dispersed food resources (Wright and Gompper 2005). Furthermore, although the raccoon populations had similar endoparasite prevalence and abundance before supplementation, both parasite metrics increased in the population with clumped supplemental food resources due to increased contact rates among individuals (Wright and Gompper 2005). The population size of racoons did not change with resource clumping, indicating that behavioral changes per se were responsible for altering parasite dynamics in this system. Human‐augmented food sources for wildlife have also been shown to alter wildlife behavior (e.g., Prange et al. 2004; Yirga et al. 2012), increasing contact rates within and between species (Totton et al. 2002; e.g., Campbell et al. 2013), and have been linked to altered disease outbreaks in a number of systems (e.g., Totton et al. 2002; Hosseini et al. 2004; Cross et al. 2007). Although not all of these studies were conducted in a heavily modified landscape, these studies demonstrate how food augmentation (whether purposeful or unintentional) in human‐modified areas might influence host behavior, impacting disease dynamics. However, in these previous studies, elevated contact rates were identified as the dominant mechanism linking shifts in behavior with altered disease dynamics and increases pathogen transmission. Resource augmentation may also cause behaviorally induced changes that may indirectly alter disease dynamics, but these indirect mechanisms have not yet been fully explored.

Here, we examine whether human‐modified habitats such as garbage sites are associated with altered behavior and disease dynamics in banded mongooses (*Mungos mungo*), a mammal that is currently affected by an observable disease, and that readily uses both natural and human‐modified habitats for foraging. Banded mongooses are diurnal, highly social, fossorial carnivores that eat invertebrates (e.g., arthropods and worms) and small vertebrates (e.g., amphibians and small mammals) in their natural habitat (Rood 1975), but at garbage in human‐modified areas, forage predominately on discarded human food. Our study population in Botswana is infected with a novel, fatal *Mycobacterium tuberculosis* complex (TB) pathogen, *M. mungi* (Alexander et al. 2010), threatening the persistence of smaller mongoose groups (Alexander et al. 2016). Primary transmission of *M. mungi* does not occur through aerosol or oral transmission typical of most members of the *M. tuberculosis* complex. Rather, *M. mungi* invades the mongoose host through cuts or abrasions on the skin or nose and is shed predominantly through anal gland secretions used in scent marking behavior important in olfactory communication (Alexander et al. 2016). Considering the unusual route of pathogen invasion, behavioral interactions such as aggression that result in cuts or abrasions (hereafter, injuries) may be particularly important to disease dynamics in this system. Although aggressive interactions among group members are rare in this low‐skew, communal species due to a relaxed or absent dominance structure (Gilchrist 2006, Muller et al. 2008), substantial levels of within‐group aggression appear to occur at human garbage sites. We therefore quantified (1) whether aggression during foraging differed across five types of foraging habitat with varying degrees of human modification, from garbage sites to natural, unmodified environments, (2) whether higher rates of within‐troop aggression were associated with increased rates of injury, and (3) whether higher injury incidence was associated with increased incidence of clinical TB.

## Materials and Methods

### General observation methods

#### Tracking and Identification

We studied four banded mongoose troops in and around the town of Kasane Botswana, near Chobe National Park, from March to November 2011. Observed troops ranged in size from 11 to 56 individuals. One or two animals in each troop were radio‐collared as described elsewhere (Laver 2013). Individual mongooses are virtually indistinguishable from one another by appearance, regardless of sex. Therefore, ear tags were used to mark up to six additional animals in the troop. Some animals were also identifiable by natural scars or injuries. Thus, taken together at least 10–33% of the individuals within each troop were identifiable over months or years, allowing longitudinal data collection for these individuals, which included both adults and juveniles. This study was conducted under a permit from the Botswana Ministry of Environment, Wildlife, and Tourism and under approval of the Virginia Tech's Institutional Animal Care and Use Committee (Protocol number 07‐146‐FIW).

#### Behavioral observations

Two datasets were used in this analysis. The first is a 9‐month general behavioral dataset, composed of scan and focal samples of four mongoose troops. The second is a short‐term (3 month) data set designed specifically to measure differences in aggression at distinct foraging areas. All data were collected by one observer (BF).

To collect both data sets, mongoose troops were followed on foot or by car. Two troops were intensively observed in a given week, alternating troops in the morning and afternoon such that a total of five mornings and five afternoons per month were spent with each troop. The schedule was occasionally disrupted by weather or inability to find or follow a troop.

### Scan and focal sampling methods

While following a troop, both focal and scan samples (Altmann 1974) were collected according to a predetermined ethogram (Fairbanks et al. 2014) when at least half of the troop was active, that is, at least half of the individuals were engaged in behavior other than lying down or sitting still. Scan samples, which capture a snapshot of the behavior of all individuals in the troop, were collected at least 20 min apart, and were recorded by speaking the behavior of each visible individual into a voice recorder, along with the time and location of the scan. Focal samples of identifiable individuals were conducted between scan samples. Focal data were collected on a smartphone (Motorola Q9 h (Motorola Mobility Inc., Libertyville, IL) running Windows Mobile 6 (Microsoft, Redmond, WA)) using the program PhoneRecord (W. Tietjen, Bellarmine University, Louisville, KY), which time stamps (to the second) each key pressed on the smartphone, each of which represents a behavior from a predetermined ethogram. The order by which individuals were focal sampled was determined both randomly and opportunistically, as described elsewhere (Fairbanks et al. 2014). Each focal sample was between 1.5 and 10 min long. If an animal went out of sight during a sample, the sample was re‐commenced when the animal came back into sight as long as the entire sample fell within 10 min. During focal and scan samples, a behavior was scored as aggression if individuals made agonistic physical contact with one another, including lunging contact, biting, scratching, or deliberate and forceful body contact.

### Foraging aggression methods

To collect targeted data on aggression in different types of foraging areas, we counted the number of aggressive sounds emitted each minute while a troop was foraging. Aggressive sounds were measured rather than aggressive behaviors because all animals in a group cannot be seen simultaneously (particularly when they forage in garbage, where garbage containers and garbage itself can obstruct observation), but all can usually be heard. Furthermore, during scan and focal samples, we observed that nearly all incidents of aggression between mongooses were accompanied by an aggressive sound. Aggressive sounds, therefore, serve as a reasonable index for aggressive behaviors. Aggressive sound data were collected on 1 or 2 days of each of 3 months (August, September, October) within the intensive observation schedule described above.

We measured aggressive sounds during banded mongoose foraging at five types of foraging locations: (1) at garbage, including household garbage bins, larger garbage receptacles, or areas of ground where humans regularly discarded garbage; (2) under outdoor lights, which attract insects at night that might die or burrow into the soil under the lights, allowing mongooses to forage on them during the day; (3) on lawns, which might draw mongooses' natural prey due to daily watering and thick grass cover (in contrast to the surrounding dry natural landscape during the study period); (4) in human‐modified areas that do not have predictable sources of food for mongooses, such as roads and paths (these areas control for effects of resource clumping found at garbage and are hereafter called “other modified areas”); and (5) in their natural habitat (while few places on earth are truly unaltered by humans, we are considering areas such as Chobe National Park and other primarily undeveloped areas to be natural habitat).

### Disease and injury observations

In addition to the behavioral observations described above, signs of TB and injury were assessed each day by observing as many individuals as possible through binoculars and/or at close range. Clinical signs of TB include cachexia, hunched body posture, matted fur, epiphora, sneezing, rhinorrhea, nasal enlargement, deviation of the nasal septum, drooping and/or enlarged testicles, lethargy, lagging behind the group, and fearlessness (Alexander et al. 2016). This clinical presentation has only been associated with *M. mungi* infection over 15 years of observation in the study area (Alexander et al. 2016) and was used to clinically characterize mongoose as being diseased or healthy. This syndromic approach to observational health classification as has been employed in other systems where a visible and specific clinical presentation is predictive of pathogen infection (e.g., *Mycoplasma gallisepticum* infection in house finches; Altizer et al. 2004). Signs were graded on a scale from 1 to 10, with 10 being completely healthy and 1 being the most severe clinical presentation. For data analysis, we defined an individual as diseased if, during the last month it was observed, it displayed at least three clinical signs rated as eight or less. Where necropsies were later conducted, mongooses with this rating were invariably found positive for *M. mungi* infection. The start month for each disease case was defined as the first month that a diseased individual displayed two signs rated nine or less.

We defined injury as any break in the skin, swelling, or limping, persisting for more than 5 days. Injuries were described and if possible, scores were given on the same type of scale as clinical signs (e.g., limping was a common injury and could be scored from a very mild, weight‐bearing limp, 9, to the animal dragging the limb and bearing no weight on it, 1).

### Data analysis: is aggression higher in human‐modified foraging habitats?

Using the foraging aggression dataset described above, a generalized linear mixed model (GLMM) was used to determine whether mongooses utilizing the different foraging area types differed by the number of aggressive sounds they emitted. The number of aggressive sounds during a foraging bout was the response variable and area type was the fixed effect. Because the number of observation minutes and troop size varied by troop and foraging bout, we used log (minutes*troop size) as an offset in the model. For our data, this offset, a statistical tool used to account for unequal explanatory variables (such as observation time) across subjects, is akin to analyzing on a “per minute per individual” basis. Troop was included as a random effect. We used a negative binomial distribution with a log link function, and the Satterthwaite method to determine denominator degrees of freedom. Because of overdispersion (*χ* ^2^/df = 3.41), an R‐side scale parameter was used (Bolker et al. 2009). The Tukey–Kramer adjustment was used as a post hoc test to determine whether different areas had significantly different levels of aggression from one another.

### Data analysis: does aggression predict injury?

We next examined whether troop‐level rates of aggression were associated with rates of injury in a troop. We used monthly totals per troop to account for the nonindependence of observations made on the same troop during the same month. Because of the limited sample size inherent in our foraging aggression dataset when summarized monthly, we utilized our more extensive focal sample dataset to determine whether aggression had an effect on the number of injuries in a troop over a longer period of time. Aggression tends to be very brief in mongoose troops (rarely lasting more than 1 sec according to our focal sample data). Therefore, we used focal rather than scan samples for this analysis because they reliably capture these brief events. To analyze the data we totaled the number of injuries, the number of aggressive behaviors observed during focal samples, and observation time (i.e., the total number of minutes of focal samples) per month for each troop (*n* = 34 troop‐months). We performed a GLMM with number of injuries as the response variable, and aggression per minute of observation, troop size, and month (class variable) as fixed effects (all 2‐way interactions were not significant and were removed from the final model). Troop was included as a random effect. We expected autocorrelation in number of injuries from 1 month to the next, so we used first‐order auto‐regression (AR(1)) to account for autocorrelation between months within troops. We used a negative binomial distribution with a log link function, and there was only slight overdispersion (*χ* ^2^/df = 1.05). We used the Satterthwaite method to determine denominator degrees of freedom.

### Data analysis: does injury predict tuberculosis?

To determine whether injured animals were more likely to present with clinical signs of TB than uninjured animals, we compared the proportion of visibly uninjured mongooses that showed clinical signs of TB across all observed troops with the proportion of injured healthy mongooses that progressed to clinical TB over a minimum observational period using a likelihood ratio chi‐square test. We only included study animals in this group that could be monitored for a minimum of 5 months postinjury. This time frame was identified to ensure a sufficiently large observational period that would exclude those individuals that disappeared or died shortly or within a few months after injury, potentially before infection may have had time to progress to clinical disease. All analyses were performed using SAS 9.3 (Cary, NC).

## Results

### Aggression rates across foraging habitats

**Figure 1:**
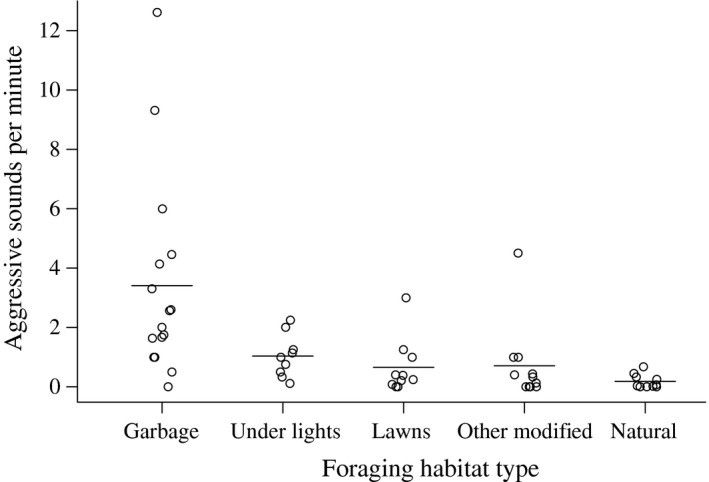
The number of aggressive sounds emitted by banded mongooses per minute during each foraging visit by area type. Horizontal lines are means for each foraging habitat type, and types are ordered by degree of human modification, decreasing from right to left.

The extent of aggression varied significantly by area type (*F*_4,51_ = 10.63, *P* < 0.0001, Fig. 1), with the highest levels of aggression at garbage sites. The parameter estimate for aggression at garbage was significantly different from zero (*t* = 2.93, *P* < 0.0001) and the least squares (LS) mean of garbage was significantly greater than lawn, natural, and other modified areas, and nearly significantly different from areas under lights (Table 1). The parameter estimate for areas under lights was significantly different from zero (*t* = 2.46, *P* = 0.01), but its LS mean was not significantly different from the other area types (Table 1). The parameter estimates for lawn, other modified areas, and natural areas were not significantly different from zero, and their LS means were not significantly different from one other (Table 1).

**Table 1:** Parameter estimates and *t*‐values for differences in aggression in five foraging habitat types

Area	Parameter estimate (SE)	*t*‐Value (*P*‐value) for parameter difference from zero	*t*‐Values (*P*‐values) for differences of least squares means				
			Garbage	Light	Lawn	Other modified	Natural
Garbage	2.92 (0.52)	5.61 (<0.0001)		2.66 (0.08)	4.34 (<0.01)^b^	4.11 (<0.01)^b^	5.61 (<0.0001)^b^
Under lights	1.47 (0.60)	2.46 (0.01)			0.96 (0.87)	1.37 (0.65)	2.46 (0.12)
Lawn	0.89 (0.58)	1.54 (0.13)				0.48 (0.99)	1.54 (0.54)
Other modified	0.60 (0.63)	0.94 (0.35)					0.94 (0.88)
Natural	0^a^						

a In proc GLIMMIX, one parameter estimate (here, natural) is set to zero to estimate the other parameters.

b Indicates significant differences.

### Aggression rates and injury

Using the dataset of focal samples across 9 months of observation, troop‐level aggression significantly predicted injury counts (*F*_1,20.18_ = 13.06, *P* = 0.002), as did month (*F*_8,19.36_ = 8.11, *P* < 0.0001). Troop size was not a significant predictor of troop‐level injury rates (*F*_1,19.45_ = 2.84, *P* = 0.11).

### Injury and TB

Of the injured animals that did not die, heal, or disappear within a day or two of injury (*n* = 29), 12 were observable for at least 5 months after injury. Of these 12, nine advanced to clinical TB within 5 months (75%, 95% CI = 50–100%) a proportion significantly higher than the proportion of clinical TB cases identified in visibly uninjured mongooses in 2011 (0%, *n* = 126; LR *χ* ^2^ = 53.04, *P* < 0.0001). In other words, in 2011 all cases of clinical TB in the observed troops were preceded by a visible injury. Injuries associated with progression to TB disease presented as persistent, nonhealing lesions and/or limping for >2 months duration.

## Discussion

Here we show that banded mongooses exhibited significantly higher rates of within‐troop aggression while foraging in garbage, where resources are clumped and likely of high value (i.e., calorie‐dense or large food items). Furthermore, troops with higher rates of aggression had higher numbers of injuries, and injuries were significantly associated with the appearance of clinical TB within 5 months. The strong association between injury and TB disease is consistent with pathogen invasion occurring through breaks in the skin or nasal planum (Alexander et al. 2016). Together, the combined associations among aggression at garbage, injury, and disease suggest that augmented food resources, a common feature of human‐modified habitats (Shochat et al. 2006), may cause changes in mongoose behavior in ways that might indirectly affect TB dynamics. Although experimental manipulations are needed to confirm causation, our results suggest that increased aggression at garbage may result in injuries that allow an environmentally associated pathogen to invade the mongoose host. Given the unique transmission mode of *M. mungi wherein the pathogen is shed via urine or anal gland secretions and invades through breaks in the skin* (Alexander et al. 2016), gathering to forage at garbage does not result in conspecifics directly passing the pathogen to each other while aggregated at garbage sites, as was detected in a study of racoon parasites at clumped augmented resources (Wright and Gompper 2005). Furthermore, in this scenario the garbage itself is not a source of infection, as was the case with baboons that became infected with bovine TB (Sapolsky and Share 2004). Instead, this system may represent a unique example whereby garbage sites indirectly modify disease transmission dynamics by altering mongoose behavior in ways that facilitate injury and subsequent pathogen invasion.

The significantly higher levels of aggressive behavior that we observed in banded mongooses foraging at garbage (Fig. 1, Table 1) are most likely primarily due to the highly clumped and presumably valuable nature of food resources found at these sites. While aggression is undoubtedly influence by other unmeasured factors (e.g., number of food items, hunger level, etc.) this behavior appeared to occur most often in response to the discovery of large food items regardless of foraging habitat type. However, if mongooses were dispersed enough to allow the individual that found the item to remain undetected by the troop, then aggression was less likely. Thus, differences in aggression during mongoose foraging may be due largely to an interaction between item size (perceived value) and resource distribution, although further research is needed to specifically address this question. While visual obstructions might help a mongoose keep a large food item out of sight of conspecifics even in a clumped foraging area such as garbage, mongooses typically give a specific, medium‐volume vocalization while eating that can draw the attention of nearby conspecifics even if the eater is not visible. This vocalization in addition to mongooses' excellent sense of smell, likely makes resource distribution more important than visual obstruction when trying to consume a high value item without needing to defend it.

Our “other modified” habitats, such as paths, that infrequently have large food items, served as a control for the possibility that human modification itself, rather than resource clumping or food item size, alter banded mongoose aggression. That aggression levels in the “other modified” habitats were similar to those observed in natural habitats supports the interpretation that variation in resource clumping and/or quality at garbage is the likely mechanism driving our results. There was also a nonsignificant trend for mongooses to be somewhat more aggressive while foraging for insects under lights than while foraging on lawns, other modified areas, or their natural habitat (Fig. 1). While insects are a natural part of the mongooses' diet, they are likely to be more highly clumped below lights than in their natural environment. Further work should be directed at measuring the extent to which banded mongoose resources are clumped and perceived as valuable on lawns, under lights, and at garbage, to confirm the role of resource concentration versus food item value in driving the detected results. More broadly, these results suggest that researchers should consider multiple forms of human modification when investigating its effects on wildlife.

Aggression associated with human habitat alteration is particularly important in understanding disease transmission dynamics as it can influence both pathogen exposure and susceptibility (Hawley et al. 2011; Fairbanks and Hawley 2012). Not only can wounds be a potential site for exposure to pathogens in the environment, as appears to be the case in our study system, but pathogens can also be directly transmitted during aggressive behavior in some systems (e.g., McCallum et al. 2007). In addition, aggression can cause neuroendocrine responses, such as changes in glucocorticoid (a.k.a. stress hormone) levels, that may lead to changes in immunity and pathogen susceptibility (e.g., de Groot et al. 2001). Evidence to date does not suggest an association between aggression and glucocorticoid levels in this system: mongoose troops at our study site with access to garbage typically have lower glucocorticoid levels (as measured by fecal glucocorticoid metabolite analysis) than those without access (Laver et al. 2012; Laver 2013). This lack of relationship between glucocorticoids and garbage access suggests that exposure, rather than susceptibility, may be the most important mechanism linking aggression and disease via injury in banded mongooses. However, changes in aggression due to human modification may affect both exposure and susceptibility simultaneously in some systems, causing substantial alterations to disease spread.

Our research underscores the need to examine the indirect effects of landscape modification on disease dynamics beyond influences on population dynamics of pathogens, vectors, and hosts. Behavioral changes may work synergistically with (e.g., Plowright et al. 2011), antagonistically to (Page et al. 2008), or in lieu of (Wright and Gompper 2005) changes in population characteristics that lead to changes in disease dynamics. These interactions have important applied implications, because culling or otherwise reducing wildlife populations is frequently considered an important first option for disease control. Reducing host population size may not have the desired effect if disease dynamics are heavily influenced by behaviors that are not density dependent. When trying to understand or mitigate changes to disease dynamics caused by urbanization and other forms of habitat modification, studies should consider behavioral and potential physiological changes alongside the better studied population‐level parameters such as host density. Our study suggests that, for some systems, property managers may have the opportunity to reduce their impact on wildlife diseases using relatively simple practices, such as excluding wildlife from garbage at residences and businesses.

## Data Accessibility

Data will be accessible on Dryad.

## Conflict of Interest

None declared.
